# School Leadership Enhances Secondary Students’ Achievement in Rural China Through Teacher Commitment and Collaborative Culture

**DOI:** 10.3389/fpsyg.2022.894688

**Published:** 2022-05-23

**Authors:** Ling Li, Haixue Zhu, Hui Li

**Affiliations:** ^1^Center for Education Policy, Southwest University, Chongqing, China; ^2^College of Educational Science, Chuzhou University, Anhui, China; ^3^Macquarie School of Education, Macquarie University, Sydney, NSW, Australia; ^4^Institute of Early Childhood Education, Shanghai Normal University, Shanghai, China

**Keywords:** school leadership, teacher commitment, collaborative culture, student achievement, moderated mediation model

## Abstract

The effect of school leadership (SL) on student achievement (SA) has been extensively examined, whereas the influences of teacher commitment (TC) and collaborative culture (CC) have not been thoroughly explored. This study conducted a moderated mediation analysis by investigating (a) TC as a mediator in the relationship between SL and SA and (b) CC as a moderator of the relationship between SL and SA. Altogether, 3,134 (female =1,673, 53.4%; male =1,461, 46.4%) students and their 841 teachers from 80 middle schools in rural China were recruited and surveyed. SA was evaluated using Program for International Student Assessment (PISA) 2008 tests, including reading, math, and science, and SL, TC, and CC were evaluated using the Teaching and Leading in Schools Survey Scale. In addition, the “many to many” step was employed to match teachers’ data with the students’ data by STATA analysis. The results indicated that: (1) there were direct and indirect effects of SL on SA in the mediation model; (2) TC was confirmed as a full mediator between SL and SA; and (3) CC acted as a significant moderator of SL effects on SA through TC. Implications for improving school leadership and student achievement are discussed.

## Introduction

Research linking school leadership (SL) to student achievement (SA) has been challenged by two difficulties—data availability and the complex nature of SL work (Grissom et al., 2013). To overcome the first difficulty, researchers have tried to collect data on a vast array of organizational processes and have just confirmed that school leadership largely acted as an indirect factor, which was mediated by organizational and teacher factors (Sebastian and Allensworth, 2012). To tackle the second challenge, scholars have explored many aspects of school leadership work, such as instructional leadership, organization management, school climate, and professional development (Heck and Hallinger, 2009; Barnes et al., 2010; Camburn et al., 2010; Grissom et al., 2013). However, very few studies have explored the mediating effects of teacher factors such as teacher commitment (TC) and collaborative culture (CC) between SL and SA. Furthermore, most existing studies have been conducted in urban schools in European-American societies, leaving those rural schools in developing countries under-studied. This study endeavors to examine the mediating or moderating roles of teacher commitment and collaborative culture with the data collected from schools in rural China.

### School Leadership and Student Achievement

School leadership refers to the process that school leaders develop vision and missions for schools, motivate and develop teachers to realize the vision and goals, build up a conducive school culture, and promote the interaction between the whole school staff, students, and other stakeholders (Zhang, 2007; Leithwood and Sun, 2012; Bush and Glover, 2014). In this study, the school leadership has four major domains: setting directions, designing the organization, improving the instructional program, and developing people (Leithwood et al., 2010b, 2019a; Leithwood and McCullough, 2016). It has been widely accepted that school leadership plays a critical role in managing school and improving the quality of learning and teaching (Sebastian and Allensworth, 2012; Zhang, 2013). Recently, school leadership has been found to be the second most influential factor associated with student achievement after teaching influence (Leithwood et al., 2008, 2020). Based on large-scale qualitative and quantitative data, the synthesis of previous research evidence, and a series of meta-analyses, Leithwood and his associates identified four domains of successful leadership practices that are helpful in schools: leadership practices related to setting directions, developing people, redesigning the organization, and improving the instructional program (Leithwood, 2010; Leithwood and McCullough, 2016; Leithwood et al., 2019b). Similar findings have also been reported in other studies (Witziers et al., 2003; Robinson et al., 2008; Heck and Hallinger, 2009; Chang, 2011; Shatzer et al., 2013; Sun and Leithwood, 2015; Wu et al., 2019). Generally, school leadership accounts for about 3–5% of the variances in student achievement (Hallinger and Heck, 1998; Leithwood et al., 2010b). This study will test this hypothesis that school leadership directly affects student achievement with rural Chinese students’ data.

However, scholars have also realized that the influence of school leadership on student achievement is a complicated process and is mostly indirect (Leithwood et al., 2010a; Sebastian and Allensworth, 2012; Day et al., 2016). Therefore, multiple research methods have been used to tease out those indirect influences, such as indirect effect, mediated effect, and moderate effect, to unveil the interaction between school leadership and school environments (Hallinger and Heck, 1998; Gates et al., 2006; De Maeyer et al., 2007; Holloway, 2013; Zhu et al., 2020). The most comprehensive model developed in this direction of studies is probably the Four Path model developed by Leithwood and his associates (Leithwood et al., 2010a, 2019b; Sun and Leithwood, 2015). This model demonstrates the four paths from SL to SA: Rational, Emotional, Organizational, and Family. Each path connects the key conditions or variables that can be influenced by those exercising leadership and will have relatively direct effects on students (Leithwood et al., 2019b). The Emotional Path, for example, includes those feelings, dispositions, or affective states of teachers (both individually and collectively) shaping the nature of their work. And teacher commitment, collective efficacy, and trust are the three significant contributors to student learning that might have blocked this path (Leithwood et al., 2019b). Therefore, this study will explore the mediating effect of teacher commitment and the moderating role of collective culture between school leadership and student achievement in rural Chinese schools.

### Teacher Commitment

Teacher commitment refers to the mental pattern that teachers firmly believe in school goals, identify with and accept school values, do their best for the school spontaneously, and aspire to work in the school continuously (Song and Cai, 2005). It is important for school reform and improvement (Yang et al., 2019). The existing studies have identified the four dimensions of teacher commitment: commitment to teaching (Firestone and Rosenblum, 1989; Billingsley and Cross, 1992; Menzies, 1995; Gordon, 1999); commitment to students (Firestone and Rosenblum, 1989; Nir, 2002); commitment to the organization (Porter et al., 1974; Freeston, 1987; Leithwood et al., 1999); commitment to change (Leithwood et al., 1999).

Previous studies have indicated a significant positive relationship between TC and SA (Firestone and Pennell, 1993; Harvey et al., 1998; Housego, 1999; Pranita, 2018; Cansoy et al., 2020). Raising the level of teacher commitment can improve students’ academic performance (Ingersoll, 2001). In particular, the first three dimensions of teacher commitment (teaching, students, and schools) are positively related to student learning (Harvey et al., 1998; Gill and Reynolds, 1999; Housego, 1999; Langer, 2000; Glaze, 2001; Griessler, 2001; Strahan et al., 2001; Johnson et al., 2012). Furthermore, some studies have confirmed that TC has mediating effects on the relationship between SL and teacher teaching (Ross and Gray, 2006; Liu et al., 2016; Piyaman et al., 2017; Hallinger et al., 2018; Liu and Hallinger, 2018).

However, school leaders can influence teachers’ commitment through the interaction of their values, motives, personality, understanding, and attitudes and those of the teachers (Sun, 2004). In particular, principals can enhance teacher commitment to the school by decision-making and continuous improvement (Robinson et al., 2008). And school leadership can affect teacher motivation and teaching quality, which subsequently impacts student achievement (Sergiovanni, 2001; Marzano et al., 2005; Leithwood et al., 2010b). Besides, many studies have reported unanimously that school leadership was significantly correlated with teachers’ willingness to make extra efforts in teaching (Leithwood and Sun, 2012). Unfortunately, in China, rural schools usually have low-achieving and low SES students; thus, they are often neglected by those studies on the impact of teacher commitment on academic achievement. This study will fill this gap and examine the hypothesis that teacher commitment mediates the effect of school leadership on student achievement.

### Collaborative Culture

Collaborative culture is an environment where staff members work together in interdependent teams that pursue common goals (Eaker and DuFour, 2009). In this study, collaborative culture refers to adjusting the instruction based on feedback from other colleagues in the school or outside the school, challenging colleagues’ beliefs about education, collaborating on teaching and learning, and celebrating the achievements of staff and students (Leithwood, 2017; Leithwood et al., 2019a). Collaborative culture and school structures could be influenced by school leadership and could jointly impact teaching and learning (Lomos et al., 2011; Camburn and Han, 2017). Previous studies have proved that collaborative culture built by school leaders could improve teachers’ professional development, job satisfaction, and commitment (Eaker and DuFour, 2009; Hong et al., 2016; Olson, 2019; Suhardi et al., 2019). For example, Olson (2019) found that school leadership could positively influence teacher commitment by building a collaborative culture. The higher collaborative culture created by school leaders, the more teachers feel the development and growth of the school; and accordingly, the more recognizing of the school, the more willing to pay more effort on school development, and eventually, the higher level of teacher commitment (Li, 2016). Similar findings were also found by Beattie (2002). In contrast, teachers who experience the low collaborative culture of the school will lose enthusiasm and interest in teaching. Their work efficiency will also be significantly reduced, which will harm students’ learning. It indicated that the collaboration among teachers was directly related to teacher commitment (Ibrahim et al., 2014). Brandon et al. (2018) also found that working collaboratively within teams would improve teaching and increase success rates so that they would devote more and more energy and commitment to schools. Therefore, this study will examine the hypothesis that collaborative culture moderates the effect of school leadership on teacher commitment.

### The Theoretical Framework of This Study

**Figure 1:**
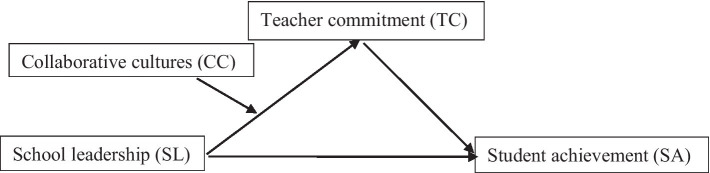
Proposed moderated mediation model linking SL to SA in rural Chinese schools.

This study was guided by the Ontario Leadership Framework (OLF) developed by Leithwood and his team (Leithwood, 2012) and its underpinning theory—the transformational leadership theory. OLF has extended the concept of transformational leadership to more elements: building the school vision and goals, providing intelligence, providing personalized support, shaping the professional practice and values, setting high-performance expectations, promoting the school decision-making, monitoring the school teaching activities, linking with the community, etc. (Leithwood, 1994; Leithwood and Jantzi, 2000; Leithwood and Sun, 2012). According to transformational leadership theory, school leadership was approaching the practice of real leadership. Also, transformational leadership has been regarded as an ideal leadership style for school leadership (Leithwood and Jantzi, 2006). Therefore, this study endeavors to examine the mediating or moderating roles of teacher commitment and collaborative culture with the data collected from schools in rural China based on the theory of transformational leadership and the Ontario Leadership Framework (see Figure 1).

In this study, data were collected from 3,134 students and their 841 teachers involving 80 middle schools in rural China. A moderated mediation model was used in this study to test school leadership effects on student achievement, where teacher commitment acted as a mediator and collaborative culture as a moderator. This study will make three contributions to research that examines how school leadership contributes to student achievement. First, it will expand the theory of school leadership beyond the European-American contexts using the data from rural schools in China. Second, it will explore how teacher commitment serves as an underlying psychological mechanism through which school leadership promotes student achievement in rural schools. Third, it will demonstrate how variation in collaborative culture can moderate the mediating effects of teacher commitment in the path from school leadership to student achievement. Accordingly, this study would examine the following hypotheses:

*Hypothesis 1*: School leadership directly affects student achievement.

*Hypothesis 2*: Teacher commitment significantly mediates the effects of school leadership on student achievement.

*Hypothesis 3*: Collaborative culture significantly moderates the mediating effect of teacher commitment between school leadership and student achievement.

## Materials and Methods

### Participants

Whole group sampling was used to investigate 102 rural junior middle schools from C Province in western China. Students (*M* = 15 years, ranged between 14 and 17 years) from the same class of junior middle schools in the surveyed schools were randomly selected as the samples. On the one hand, we collected data on the basic information of the school students and student achievement through questionnaires and field observation. In this step, the Program for International Student Assessment (PISA) exam results were used to measure student achievement. On the other hand, we collected variable data from teachers on school leadership, teacher commitment, and collaborative culture to explore how school leadership provides important support influence on student achievement. In this step, the class teachers of all the participating students were invited to complete an anonymous survey on school leadership, teacher commitment, and collaborative culture. Altogether 1,245 teachers consented to participate in this study and returned the completed questionnaire with the sealed envelopes provided by the researchers. In addition, we matched the students with their teachers using the school name as the matching identifier. And “many to many” step of STATA analysis was conducted to do the matching. Eventually, 841 teachers from 80 classes/schools were successfully matched with their 3,134 students (female = 1,673, 53.4%; male = 1,461, 46.4%), resulting in a teacher-student ratio of 1:2.5 that is appropriate for controlling the nested effects in school data.

### Measures

#### Student Achievement

Student achievement was assessed using the Chinese version of the PISA exam. An internationally standardized academic achievement test organized by the Organization for Economic Co-operation and Development (OECD), PISA aims to evaluate the academic performance of 15-years-old in the subject of reading, math, and science. Its Chinese version was first applied to the 15-years-old in Shanghai in 2009 (Sellar and Lingard, 2013) and later released online for public use.^1^ The raw score for each student was converted to a standardized score (z score) in the statistical analysis.

#### School Leadership

The Teaching and Leading in Schools Survey Scale (Leithwood, 2017) was translated into Chinese and adopted to measure SL. This questionnaire has four constructs and 20 items. The teachers were asked about the extent to which they believed their school leadership was: “Provide useful assistance to your staff in setting short-term goals for teaching and learning,” “Develop an atmosphere of caring and trust with your staff.” School leadership was assessed by a five-point Likert scale which scored from 1 (not at all confident) to 5 (very confident). The psychometric properties of this Chinese version are very satisfactory: Cronbach *α* was 0.983, and the half-scale reliability was 0.977.

#### Teacher Commitment

Teacher commitment was measured using the Chinese version of *the Teaching and Leading in Schools Survey Scale* (Leithwood, 2017), five items on TC. For example, “I am willing to ‘go the extra mile’ to help my school achieve its goals for our students,” “I refine my instructional strategies based on evidence of how well my teaching contributes to my school’s goals for our students.” TC in this study was assessed using a Likert scale: 1 = strongly disagree to 5 = strongly agree. It was found a useful tool with satisfactory reliability and validity for assessing teacher commitment in this study. Cronbach *α* was 0.915, and the half-scale reliability was 0.900.

#### Collaborative Culture

Collaborative culture was measured using the Chinese version of *the Teaching and Leading in Schools Survey Scale* (Leithwood, 2017), which also has nine items on CC. For example, “We collaborate with one another to develop common assessment tools for measuring students’ progress,” “We frequently discuss how to best implement our improvement initiatives.” CC in this study was assessed using a Likert scale: 1 = strongly disagree to 5 = strongly agree. It was found a useful tool with satisfactory reliability and validity for assessing CC in this study. Cronbach *α* was 0.930, and the half-scale reliability was 0.886.

### Control Variable

Gender and registered residence of students (RRS) and teaching ages of teachers (TAT) were acted as the control variable in this study.

### Data Analysis Plan

To address the three research questions, three steps of data analysis were conducted in this study. The first step was descriptive statistics and correlation analysis. SPSS 25.0 was used to describe the overall characteristics of SL, TC, CC, SA, and control variables and analyze their correlation. The second step was the PROCESS 3.2 (Hayes, 2013) mediation analysis to explore the mediating effect of TC between SL and SA. The third step was conducted to estimate the moderating effect of CC on TC, based on the model confirmed by the second step. Accordingly, we have tested whether the influence of SL on TC varies with a different CC.

## Results

### Preliminary Analyses

Table 1 presents descriptive statistics and bivariate correlations for all the study variables. The mean scores showed: SL (*M* = 4.103, *SD* = 0.499), TC (*M* = 4.406, *SD* = 0.333), and CC (*M* = 4.151, *SD* = 0.426). The bivariate correlations results showed that there were significant correlations between SL, TC, CC, and SA (*p* < 0.05). The results are quite similar to the existing studies conducted in China (Liu and Hallinger, 2020).

**Table 1:** Descriptive statistics and Pearson correlations among study variables.

	Gender	RRS	TAT	SL	TC	CC	SA
Gender	−						
RRS	−0.039^*^	−					
TAT	0.026	−0.012	−				
SL	0.009	0.085^**^	−0.199^**^	−			
TC	−0.020	0.061^**^	−0.103^**^	0.696^**^	−		
CC	−0.013	0.099^**^	−0.252^**^	0.891^**^	0.720^**^	−	
SA	0.065^**^	0.088^**^	0.035^*^	0.093^**^	0.094^**^	0.128^**^	−
M	1.53	1.07	2.912	4.103	4.406	4.151	0
SD	0.499	0.272	0.573	0.499	0.333	0.426	0.818

* *p* *< 0.05;*

** *p < 0.01 (two-tailed)*.

Due to the answers in this study being based on a questionnaire test, there may have been an existing common deviation. To avoid common deviation, we adopted an anonymous response and reverse scoring to collect data in this study. Accordingly, we performed Harman’s single-factor test, a form of exploratory factor analysis on all the testing items. More than one factor emerged from this analysis, and the first factor explained only 28.62% (<40%) of the variance. This result suggests that the threat of common method bias was low (Zhou and Long, 2004), and the data could be used for further analysis. Also, Liu and Hallinger (2020) found that the teacher-reported self-reported variables were not socially desirable; instead, those Chinese teachers tended to offer a less biased judgment of their principals. Therefore, the common method bias might not constitute a significant problem in this study, even though the data came from a single source.

### Moderated Mediation Analyses

We conducted the moderated mediation analyses in three interdependent steps sequentially. First, we tested the effects of school leadership on student achievement. Second, we tested the partial mediation model to determine if teacher commitment was a significant mediator of school leadership effects on student achievement. Third, we tested whether collaborative culture moderated the relationship between school leadership and student achievement.

### Testing the Direct Effect of SL on SA

The first research question aimed to establish the direct effect model of SL on SA. The hierarchical regression was conducted, and the result (see Table 2) indicated that the path coefficient was 0.096 (*p* < 0.001). This result demonstrated that the direct effect of SL on SA was statistically significant and suitable for mediating effect analysis.

**Table 2:** Hierarchical regression analyses predicting student achievement (SA).

Dependent variable	Independent variable	*B*	*SE*	*β*	*t*
SA	TAT	0.077	0.026	0.054	2.976^**^
	Gender	0.108	0.029	0.066	3.708^***^
	RRS	0.251	0.053	0.083	4.692^***^
	SL	0.157	0.030	0.096	5.304^***^
TC	TAT	0.022	0.008	0.038	2.906^**^
	Gender	−0.018	0.009	−0.027	−2.111^*^
	RRS	0.000	0.016	0.000	0.010
	SL	0.470	0.009	0.704	53.662^**^
SA	TAT	0.086	0.026	0.060	3.279^**^
	Gender	0.107	0.029	0.065	3.707^***^
	RRS	0.247	0.053	0.082	4.630^***^
	SL	−0.034	0.054	−0.021	−0.630
	TC	0.061	0.065	0.025	0.936

* *p* *< 0.05;*

** *p* *< 0.01;*

*** *p < 0.001 (two-tailed)*.

### Testing the Mediated Effect of TC

The second research question aimed to assess the mediating effect of TC in the proposed model of SL effects on SA. Then, the TC variable was added based on the direct effect model, and the mediation model was established. As shown in Table 2, the regression results showed the direct predictive power of SL on TC was significant (*β* = 0.704, *p* < 0.001), and the TC on SA was not significant (*β* = 0.025, *p* > 0.05). After controlling for the correlated variables, the relationship between SL and SA was still not significant (*β* = − 0.021, *p* > 0.05). The traditional Sobel test was conducted, and the power was considered low in the study because it is difficult to satisfy the normal hypothesis. In the multiple mediation models, this limitation was even more. Therefore, we had to adopt the deviation correction percentile bootstrap method for the mediation model test, improving the statistical test power and having more obvious advantages than other methods (Fang et al., 2011).

Next, Bootstrap (model 4, sampling 5,000 times) was applied to verify this mediated model using the PROCESS 3.2 Macro for SPSS 25, to yield 95% CI of the indirect effect. SL was taken as an independent variable in the model, SA as a dependent variable, and TC as a mediate variable. The mediating effect is significant if the 95% CI of the mediating effect value does not cross 0. The bootstrap analyses further revealed a statistically significant indirect effect of SL on SA through TC (*β* = 0.064, *SE* = 0.026, 95% CI = [0.013, 0.116], not crossing zero). And the mediating of TC has explained 41.8% of the overall effects of SL and SA in this study. Thus, the results support a mediated relationship between SL and SA, wherein TC is a meaningful mediator.

### Testing the Moderated Effects of CC

The third research question aimed to determine if high or low CC influenced the mediation effects of TC. Again, all predictive variables were standardized, and control variables were controlled in each equation. In this moderation model, SL was taken as an independent variable, TC as a dependent variable, and the product of SL and CC was used as an interaction term to test the moderating effect. Bootstrap (model 7, sampling 5,000 times) was applied to verify this moderation model using the PROCESS 3.2 Macro for SPSS 25, to yield 95% CI of the indirect effect. When the moderating effect was significant, we took the mean plus one SD as the high group, while the mean minus one SD as the low group to draw the simple effect diagram.

**Figure 2:**
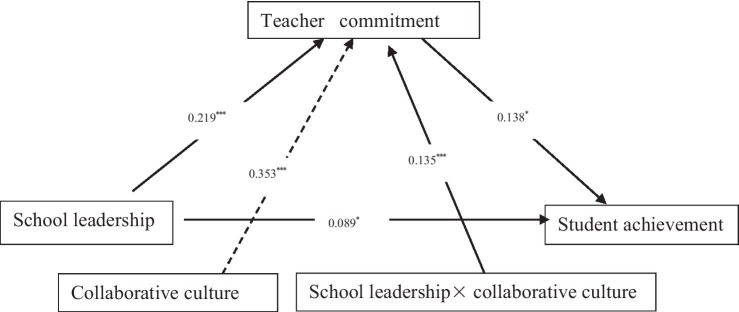
Confirmed moderated mediation model linking SL, TC, CC, and SA. *p < 0.05; ***p < 0.001 (two-tailed).

First, the results indicated that the direct predictive power of SL on TC (*β* = 0.219, *p* < 0.001), CC on TC (*β* = 0.353, *p* < 0.001), product of SL and CC on TC were all significant (*β* = 0.135, *p* < 0.001). In addition, this test demonstrated the CC had a significant positive moderating effect on the positive relationship between SL and TC (*β* = 0.019, *SE* = 0.008, 95% CI = [0.004, 0.036], not crossing zero) (see Figure 2; Table 3).

**Table 3:** Unstandardized coefficients for testing main effects and moderation effects.

	TC	SA
SL	0.219^***^ [0.178, 0.258]	0.089^*^ [0.014, 0.164]
CC	0.353^***^ [0.314, 0.392]	−
SL × CC	0.135^***^ [0.100, 0.178]	−
SA	0.138^*^ [0.027, 0.250]	−

*95% bias-corrected CIs reported as: [lower limit CI (LLCI), upper limit CI (ULCI)]*.

* *p* *< 0.05;*

*** *p < 0.001 (two-tailed)*.

**Figure 3:**
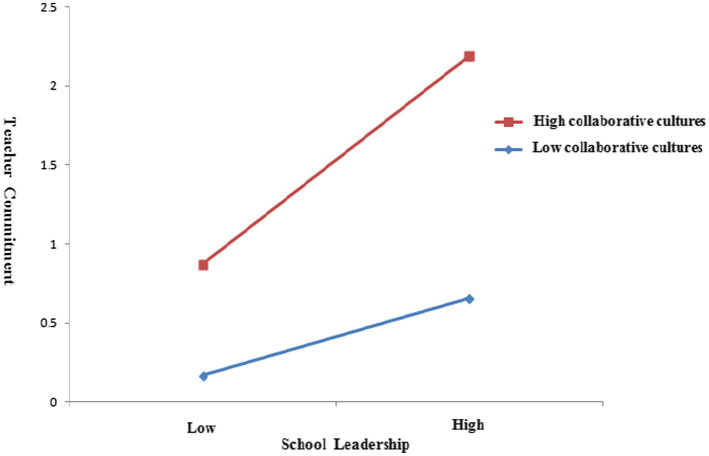
Interaction between SL and CC with regard to TC.

Second, we plotted the relationships among the three variables included in this analysis in Figure 3: CC, SL, and TC. This analysis showed that SL had a more positive association with TC when CC is higher than when it is lower. It means when CC with more cooperation between principals and teachers, the SL had a more positive impact on their commitment.

Third, to reveal the manifestation of the interaction effect more clearly, we investigated the prediction of high and low CC on the relationship between SL and TC. The mean value of the CC variable plus one SD was taken as a high CC group, and the mean value minus one SD was taken as a low CC group, respectively. The results (see Table 4) indicated that when the CC was at a higher level, the promotion effect of SL on TC was significant (*β* = 0.281, *SE* = 0.021, *p* < 0.001, 95% CI = [0.240, 0.322], crossing zero). When the CC was at a lower level, the predictive effect was significant but weakened (*β* = 0.162, *SE* = 0.018, *p* < 0.001, 95% CI = [0.127, 0.197], crossing zero). All these results indicated that when the CC was higher, the indirect effects of SL were stronger. Therefore, CC could be confirmed as the moderator of the SL mediation model. And the results support the view that higher CC could enhance SL effects on TC and SA.

**Table 4:** Conditional indirect effects of school leadership (SL) on student achievement (SA) moderated by collaborative culture (CC).

Groups	Estimate	***SE***	95% LLCI	95% ULCI
+1 Standard deviation	0.281	0.021	0.240	0.322
Mean	0.222	0.018	0.186	0.258
−1 Standard deviation	0.162	0.018	0.127	0.197

*95% bias-corrected CIs reported as: [lower limit CI (LLCI), upper limit CI (ULCI)]*.

## Discussion

As an emerging topic in the field of teacher education, the complicated and dynamic interactions between school leadership, teacher development, and student achievement have attracted global attention (Heck and Hallinger, 2009; Barnes et al., 2010; Camburn et al., 2010; Grissom et al., 2013; Liu and Hallinger, 2020; Pan and Chen, 2020). This study, conducted in rural China, more specifically, for the first time, examined how CC moderates the effects of SL on TC and SA. In this section, we will review the interpretation limitations of this study and offer our implications of the findings.

### The Moderated Mediation Model of SL, TC, CC, and SA

This study has empirically explored the influence of SL, TC, and CC on SA and confirmed the mediating effect of TC and the moderating effect of a CC. The verified model helps to reveal the underlying psychological mechanism of how SL promotes SA *via* the moderated mediation of TC in rural schools. Compared with the lower CC, SL in the higher CC has a stronger predictive effect on SA through TC. The adjustment point is in the first half of the mediating path, indicating that the relationship between SL and TC depends on the CC.

These findings, *per se*, are consistent with those of the previous studies reporting the mediating effect of TC between SL and SA (Firestone and Pennell, 1993; Alam and Ahmad, 2017). School is a learning community and a social context, a strong school leader could establish a climate conducive to teaching and learning and create a positive cultural atmosphere, which would affect TC and then SA (Hallinger and Heck, 1998; Beattie, 2002; Lomos et al., 2011; Camburn and Han, 2017). A collaborative culture is a kind of positive cultural atmosphere, thus plays a significant moderating role in the pathway from SL to TC. The existing studies (Firestone and Pennell, 1993; Harvey et al., 1998; Housego, 1999) have found that TC (not the CC) plays a significant positive effect on SA. Therefore, TC to teaching and learning was found a significant contributor to SA in this study and the previous ones (Firestone and Pennell, 1993; Harvey et al., 1998; Housego, 1999; Pranita, 2018; Cansoy et al., 2020).

### The Implications for Teacher Education

The findings of this study have some implications for teacher education. First, the finding that TC played the mediating role indicates that more attention should be paid to TC. In addition, TC is one of the important variables determining whether teachers leave schools (Wang and Zhao, 2010). It is negatively related to turnover, absenteeism, burnout, emotional exhaustion, and depersonalization (Meyer et al., 2002; Jernigan and Beggs, 2005; Schmidt, 2007; Hulpia et al., 2012). Higher levels of TC are closely related to educational effectiveness (Dee et al., 2006). Therefore, improving TC should be highly prioritized by school principals. And this is particularly important in Chinese rural schools, where turnover and burnout are frequently observed. Principals can enhance TC directly by using more words of praise and encouragement and fewer words of criticism and sarcasm or practicing transformational leadership (Leithwood and Sun, 2012). Also, the principal should know every teacher’s interests, hobbies, strengths, and specialties and assign different tasks to the most suitable person to let every teacher play a role in promoting TC. In addition, previous studies have proved that distributed leadership is an important determinant of productive collaboration (Harris et al., 2013).

Second, the finding that CC plays as a significant moderator through which principals can foster TC implies that principals should try to create a positive CC. This is consistent with what was reported in earlier studies, which reported that school leaders could positively influence teacher commitment by fostering shared governance and a culture of collaboration (Beattie, 2002), professional learning communities (PLCs; Stein and Burger, 1999; Sun, 2004; Vanblaere and Devos, 2016), and participatory decision-making (Reames and Spencer, 1998). A CC is a friendly culture where school staff works collaboratively and systematically towards a shared vision developed based on evidence. Principals can also create time and structure for teachers to collaborate and foster effective teams. This way, principals can build PLCs and create a CC to promote TC and SA. Such structures would allow teachers to participate and collaborate in making decisions about teaching and learning and improve teachers’ instructional expertise, positively affecting SA (Robinson et al., 2008; Heck and Hallinger, 2010; Leithwood et al., 2019a,b; Liu and Hallinger, 2020). In the Chinese context, teachers can share teaching and research activities such as opening classes, lesson studies, lectures, or discussions to understand teachers’ teaching style and listen to teachers’ voices (Mou, 2006).

## Limitations and Conclusion

This study has several limitations. First, the cross-sectional nature of our study yielded associations among variables in the proposed model but failed to make reliable causal inferences. Future studies using a longitudinal research design will help to conduct a dynamic evaluation of the causal direction of relationships among the constructs, we investigated in this study. Second, we evaluated school leadership based on teachers’ reports, lacking a direct measure of the interpersonal leadership styles of the principals. Future studies with direct measures on the principals could supplement this teacher-reported method to provide a triangulated evaluation of SL. Third, this study was conducted in middle schools in rural China, leaving those in urban areas untouched. Accordingly, the generalization of research findings might be limited. Future studies should involve those students, teachers, and principals in urban China to crosscheck or verify the results of this study. Nevertheless, this study does have some theoretical and practical contributions to teacher education. The findings are an essential reference for those developing countries with a substantial proportion of rural areas such as India and those in Africa and South America.

This study has empirically examined the mediating role of TC moderated by CC in the relationship between SL and SA. First, we found that SL significantly and positively predicted SA and TC. Second, we confirmed that TC played a mediating role in the influence of SL on SA. Third, CC moderated the influence of SL on SA through TC. The theoretical contribution of this study might be that it has confirmed that those findings in urban schools in European-American contexts might also apply to the rural schools in China. And the moderated mediation model of TC and CC between SL and SA might be universal and cross-cultural.

## Data Availability Statement

The original contributions presented in the study are included in the article/supplementary material, further inquiries can be directed to the corresponding author.

## Ethics Statement

The studies involving human participants were reviewed and approved by the Ethical Committee, Faculty of Education, Southwest University. Written informed consent to participate in this study was provided by the participant’s legal guardian/next of kin.

## Author Contributions

LL conducted the study. HZ and HL conducted the data analysis and drafted the manuscript. All authors contributed to the article and approved the submitted version.

## Funding

This paper is supported by Decision Making Laboratory for Western Education and Human Development, National Social Science and Humanity Foundation (ZDA338), and 111 Program; National Educational Double First-Class Program at Southwest University; Key projects of Outstanding Young Talents Support Program in Colleges and Universities (gxyqZD2020107); and Youth Project of Philosophy and Social Science Foundation of Anhui Province (AHSKQ2020D176).

## Conflict of Interest

The authors declare that the research was conducted in the absence of any commercial or financial relationships that could be construed as a potential conflict of interest.

## Publisher’s Note

All claims expressed in this article are solely those of the authors and do not necessarily represent those of their affiliated organizations, or those of the publisher, the editors and the reviewers. Any product that may be evaluated in this article, or claim that may be made by its manufacturer, is not guaranteed or endorsed by the publisher.

## Footnotes

^1^ https://www.oecd.org/pisa/data/pisa2009database-downloadabledata.htm
